# Supplementary material for: Consistent Biomarkers and Related Pathogenesis Underlying Asthma Revealed by Systems Biology Approach
Source: Int J Mol Sci. 2019 Aug 19;20(16):4037. doi: 10.3390/ijms20164037 (PMC6720652; doi:10.3390/ijms20164037)
Supplement: Supplementary file 1 [file ijms-20-04037-s001.zip › ijms-566768-supplementary-XML/Supplementary Material S2-Reports of Quality control for ten datasets.zip!/Supplementary Material S2-Reports of Quality control for ten datasets/QC_report_for_GSE89809/hm.pdf]

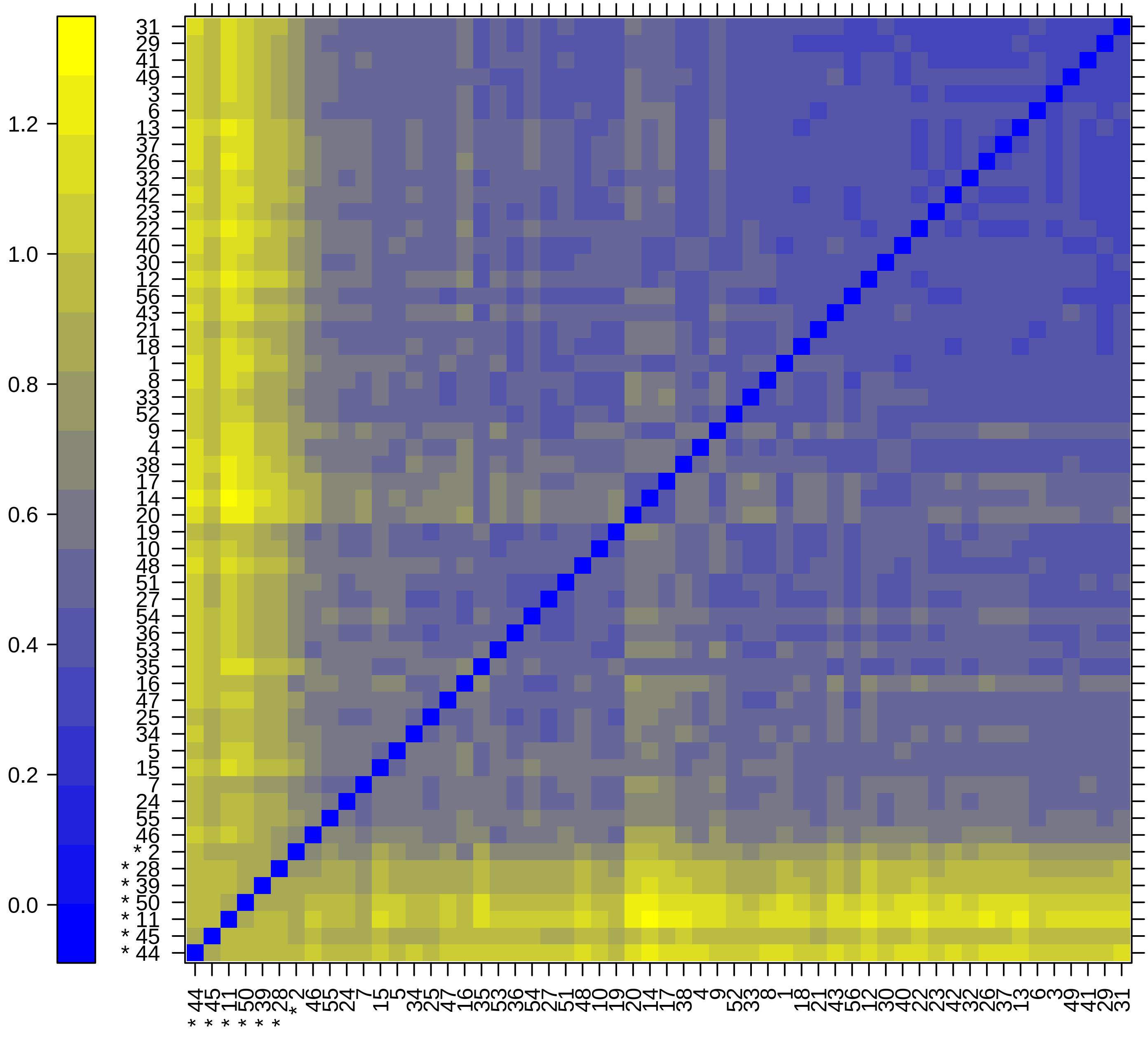
1.2
1.0
0.8
0.6
0.4
0.2
0.0
31 29 41 49 3 6 13 37 26 32 42 23 22 40 30 12 56 43 21 18 1 8 33 52 9 4 38 17 14 20 19 10 48 51 27 54 36 53 35 16 47 25 34 5 15 7 24 55 46 *2 * 28 * 39 * 50 * 11 * 45 * 44
44 45 11 50 39 28 2 46 55 24 7 15 5 34 25 47 16 35 53 36 54 27 51 48 10 19 20 14 17 38 4 9 52 33 8 1 18 21 43 56 12 30 40 22 23 42 32 26 37 13 6 3 49 41 29 31
* * * * * * *
